# Yolkin Isolated from Hen Egg Yolk as a Natural Immunoregulator, Activating Innate Immune Response in BMDM Macrophages

**DOI:** 10.1155/2020/5731021

**Published:** 2020-05-15

**Authors:** W. Kazana, M. Mitkiewicz, M. Ochnik, M. Sochocka, A. Zambrowicz, G. Piechowiak, J. Macała, P. Miernikiewicz, A. Zabłocka

**Affiliations:** ^1^Laboratory of Microbiome Immunobiology, Hirszfeld Institute of Immunology and Experimental Therapy, Polish Academy of Sciences, 12 R. Weigla Str., 53-114 Wrocław, Poland; ^2^Laboratory of Virology, Hirszfeld Institute of Immunology and Experimental Therapy, Polish Academy of Sciences, 12 R. Weigla Str., 53-114 Wrocław, Poland; ^3^Department of Functional Food Products Development, Faculty of Biotechnology and Food Sciences, Wrocław University of Environmental and Life Sciences, 37 Chełmońskiego Str., 51-630 Wrocław, Poland; ^4^Laboratory of Bacteriophages, Hirszfeld Institute of Immunology and Experimental Therapy, Polish Academy of Sciences, 12 R. Weigla Str., 53-114 Wrocław, Poland

## Abstract

One of the goals of biomedical sciences is to search and identify natural compounds that are safe, have no side effects, and possess immunostimulatory activity. It has been proven that medicines of natural origin can be effective agents, supporting the therapy of many diseases, not only in the weakened immune system of the body but also in the prevention of many diseases in healthy people. It has been shown that yolkin, a polypeptide complex isolated from hen egg yolk as a fraction accompanying immunoglobulin Y (IgY), possesses potential biological activity. However, the mechanism of its action has not been explained. The objective of this investigation was to examine the molecular mechanisms of innate immune response, activated in response to yolkin, in murine bone marrow-derived macrophages (BMDM). It was shown that yolkin induced phosphorylation of extracellular signal-kinases (ERK1/2) and c-Jun N-terminal kinase (JNK) and upregulated expression and production of type I interferons, TNF-*α* (tumor necrosis factor *α*), and nitric oxide (NO), in BMDM cells. Using pharmacological inhibitors of ERK 1/2 and JNK kinases, we revealed that the JNK signaling cascade is required for yolkin-induced inducible NOS expression and upregulation of NO production in mouse macrophages. Using the TLR4-deficient BMDM cell line, we established that yolkin can activate macrophages in a TLR4-dependent manner. It was also shown that NO, TNF-*α*, and type I IFNs (*α*/*β*) produced by BMDM cells in response to yolkin triggered antiviral activity. These data indicate that yolkin affects the regulation of the immune system and antiviral response; therefore, it can be used as an effective immunostimulator of the innate immunity or as a supplement of the conventional therapy of immunodeficiency.

## 1. Introduction

Bioactive food-derived compounds, because of their biological activity as well as their high safety profile, are essential in the development of natural drugs and nutraceuticals [1–3]. Eggs are one of the particularly rich sources of bioactive proteins and peptides, but some of them are poorly characterised. Egg yolk contains numerous vital nutrients and preservative substances, due to its original role as an embryonic chamber [1, 4]. A precursor of major proteins in the egg yolk is vitellogenin, which during egg formation is enzymatically cleaved into fragments located in yolk granules or plasma [5]. It was shown by Polanowski et al. [6, 7] that immunoglobulin Y from hen egg yolk occurs as a complex with peptides, named yolkin, exhibiting immunoregulatory properties.

Yolkin consists of several peptides with an apparent molecular weight of 1 to 35 kDa. In this mixture, polypeptides with a molecular weight from 16 to 23 kDa are most abundant. It has been shown that purified yolkin constituents are homologous with some fragments of the C-terminal domain of vitellogenin II. The fractions of MW lower than 12 kDa are free of carbohydrates, and their amino acid sequences respond to the sequence of vitellogenin II, starting from position 1732aa. Whereas fractions of MW higher than 16 kDa are glycoproteins corresponding also to the amino acid sequence of vitellogenin II starting at position 1572aa [6]. Our studies obtained up to now have demonstrated that yolkin possesses immunoregulatory, antioxidant, and neuroprotective activities, stimulating human whole blood (*ex-vivo*) to produce a wide spectrum of cytokines [6–8], downregulating the level of intracellular free oxygen radicals, and stimulating neuron-like cells to produce brain-derived neurotrophic factor (BDNF) [9].

Innate immunity is the first line of host defense that protects multicellular organisms from infectious diseases. Stimulation of the innate response is regarded as one of the most important strategies to enhance the body's defense systems, especially in elderly patients. In the elderly population, individuals become more susceptible to bacterial and viral infections and have an increased incidence of autoimmune diseases and malignancies [10, 11]. The main contributing factor to these clinical ailments is a dysfunction of the immune system. Macrophages are key cells of the innate immune system in tissue homeostasis with an active involvement in the primary immune response to pathogens, tumors, and also neurodegenerative disorders [12–14]. As professional antigen-presenting cells, they help form the innate and adaptive immune responses by producing and releasing a wide spectrum of proinflammatory mediators at an early stage of injury or infection. Macrophages commonly exist in two distinct subsets: (1) classically activated macrophages (M1), which produce proinflammatory factors like interferons (IFNs), tumor necrosis factor alpha (TNF-*α*), or nitric oxide (NO) and play an important role in the inflammatory, antiviral, and antibacterial response, and (2) alternatively activated macrophages (M2), which are anti-inflammatory and immunoregulatory, producing anti-inflammatory cytokines such as interleukin 10 (IL-10) and transforming growth factor beta (TGF-*β*). Changes in the macrophage functions enhance susceptibility to infection, tissue injury, and tumor progression [11].

Macrophages can be activated through members of the Toll-like receptor (TLR) family. The TLR4 receptor, expressed on the cell membrane of macrophages, is a crucial receptor, which recognizes patterns of bacterial polysaccharides and is also involved in the recognition of viruses by binding to viral envelope proteins [15]. Activated TLR4 triggers the activation of mitogen-activated kinase (MAPK) signaling pathways, including the c-Jun N-terminal kinase (JNK) pathways, extracellular signal-regulated kinases (ERK 1/2), and also transcriptional factor kappa B (NF-*κ*B) [16]. MAP kinases and NF-*κ*B are classic inflammation-related factors that induce the expression of proinflammatory mediators like IFNs, TNF-*α*, or inducible nitric oxide synthase (iNOS) [17].

Immunostimulation is an important strategy for enhancing the body's defense system, both in healthy people and in patients with an impaired immune response [11, 14]. Hence, looking for effective immunoregulators, specially those obtained from natural sources, which are able to activate immune cells like macrophages and T and B lymphocytes, is an important proposition of therapeutic strategies. The strategy may support the regeneration of the aging immune system and its response against a pathological environment. In the present study, the molecular mechanism of the immunoregulatory and antiviral activity of the polypeptide complex yolkin isolated from hen egg yolk, in mouse bone marrow-derived macrophages, was investigated.

## 2. Materials and Methods

### 2.1. Materials

High-glucose Dulbecco's modified Eagle's medium (DMEM), RPMI 1640 medium, and phosphate-buffered saline (PBS) (pH 7.4) were sourced from the Laboratory of General Chemistry of the Institute of Immunology and Experimental Therapy, PAS (Wroclaw, Poland). Tris (hydroxymethyl) aminomethane (Tris), Sephacryl 100-S HR resin, bacterial lipopolysaccharide (LPS) from *E. coli* (serotype 055: B5), 3-(4,5-dimethylthiazol-2-yl)-2-5-diphenyltetrazolium bromide (MTT), and Tween-20 were purchased from Sigma-Aldrich (St. Louis, MO, USA). L-glutamine and antibiotics (penicillin/streptomycin mixture) were purchased from BioWest (Nuaillé, France). Reagents for SDS-PAGE and protein markers were purchased from Bio-Rad (Hercules, CA, USA). The Mouse TNF-*α* ELISA Max™ Deluxe Kit was obtained from BioLegend (San Diego, CA, USA). N-(1-naphthyl)-ethylenediamine was purchased from Serva Feinbiochemica (Heidelberg, Germany). Sulfanilamide, sodium nitrite, orthophosphoric acid, acetone, KH_2_PO_4_, and K_2_HPO_4_ were purchased from Avantor (Gliwice, Poland). Alkaline phosphatase-conjugated anti-rabbit IgG antibody were from Cell Signaling Technology (MA, USA). Anti-ERK 1/2, anti-phospho-ERK 1/2, anti-JNK, anti-phospho-JNK monoclonal antibody, and U0126 inhibitor were obtained from Cell Signaling Technology (Leiden, The Netherlands). Anti-iNOS monoclonal antibody was from Santa Cruz Biotechnology (Santa Cruz, CA, USA). 5-Bromo-4-chloro-3-indolyl phosphate disodium salt (BCIP) and nitro-blue tetrazolium (NBT) were from Carl Roth GmbH (Karlsruhe, Germany). An endozyme test was purchased from Biomeriuex (Marcy-l'Étoile, France). The SP600125 inhibitor was from MedChem Express (NY, USA).

### 2.2. Cell Culture

The murine bone marrow-derived macrophages of the BMDM cell line and TLR4-deficient bone marrow-derived macrophages of the BMDM cell line (Rai Resources) were used in this study. The cells were maintained in Dulbecco's modified Eagle's medium (DMEM) supplemented with 10% FBS, antibiotics (penicillin, streptomycin, and gentamycin), and 3% L-glutamine. Cells were grown under standard conditions in a humidified incubator at 37°C in an atmosphere of 95% air and 5% CO_2_. Adherent cells from confluent cultures were detached, centrifuged at 150 x g for 10 min, and suspended in complete culture medium.

### 2.3. Isolation of Yolkin Polypeptide Complex

The IgY containing yolkin was isolated from egg yolks according to the procedure described in detail by Polanowski et al. [6]. Briefly, the water solution of IgY preparation was the starting material for the isolation of immunologically active peptides. The native IgY, isolated from hen egg yolk after being dialyzed for two days against two changes of 100 mM of potassium phosphate buffer, pH 7.2 and clarified by centrifugation, was chromatographed on a Sephacryl S-100 HR column (K50/100 Pharmacia Ltd., Kent, UK) equilibrated with the same buffer. The main peak of the chromatographic profile corresponded to IgY, and a small peak in some preparation tailing corresponded to low molecular weight proteins. These fractions, separated from the IgY sample named yolkin, were pooled, dialyzed against water, and lyophilized. Yolkin preparation purity was determined by SDS-PAGE. Endotoxin contamination of yolkin preparation was determined by the endozyme test, and it ruled out the presence of endotoxins in yolkin used in the present study.

### 2.4. SDS-PAGE Analysis

SDS/polyacrylamide slab gels (15%) were prepared by the use of TXG Fast Cast Acrylamide solutions (Bio-Rad, California, USA). The protein samples (10 *μ*g) were diluted with the buffer containing dithiothreitol as a reducing reagent and loaded onto gel slabs. At the end of the analysis, the gel slabs were stained with Coomassie Brilliant Blue R-250.

### 2.5. MTT Cell Viability

Cell viability was determined using the MTT colorimetric assay [18]. BMDM cells were seeded onto a 96-well plate (1 × 10^4^/well) and incubated for 24 h with yolkin preparation (10-150 *μ*g/ml). After cell treatment, the supernatants were removed and the cells were incubated with MTT (5 mg/ml) for 4 h at 37°C. The formazan crystals were dissolved by adding 100 *μ*l of DMSO and vigorously shaken to complete resolving. The absorbance was measured by an EnSpire™ 2300 microplate reader (Perkin Elmer, Massachusetts, USA) at 570 nm. Cell viability was expressed as a percentage of control.

### 2.6. Assay to Nitrite/Nitrate Generation

- BMDM cells were plated onto a 24-well plate at a density of 1 × 10^6^ cells/ml and cultured in Dulbecco's modified medium. Yolkin (10 to 150 *μ*g/ml) was added to the cells as potential inducer of nitric oxide. LPS (1 *μ*g/ml) was used as a control activator of macrophages, while untreated BMDM cells were used as a negative control. Because NO is synthetized by inducible NOS, the selective iNOS inhibitor S-MIU (10 *μ*M) was used as a relative control. Additionally, to determine the impact of ERK 1/2 and JNK kinases on the regulation of iNOS expression, BMDM cells were preincubated for 2 h with the selective kinase inhibitors U0126 (for ERK1/2) and SP600125 (for JNK) and then stimulated with yolkin (100 *μ*g/ml). After 24 hours of incubation, the supernatants were collected and the level of nitric oxide was determined.
- To determine if yolkin activates BMDM cells by the TLR4 receptor, the TLR4-deficient BMDM cell line (BMDM TLR4(-)) was used. BMDM cells were plated onto a 24-well plate at a density of 1 × 10^6^ cells/ml and cultured in Dulbecco's modified medium. Yolkin (10 to 150 *μ*g/ml) was added to the cells as potential inducer of nitric oxide. LPS (1 *μ*g/ml) was used as a classical activator of the TLR4 receptor, while untreated BMDM cells were used as a negative control. After 24 hours of incubation, the supernatants were collected and the level of nitric oxide was determined.

### 2.7. Assay to Measure Nitric Oxide (NO) Level

NO production was measured by testing the nitrite concentration in the supernatants of cultured BMDM cells using a colorimetric method with the Griess reagent [19]. In brief, 100 *μ*l samples of cell supernatants were incubated with an adequate amount of the Griess reagent (0.1% N-(1-naphthyl)-ethylenediamine and 1% sulfanilamide in 5% phosphoric acid). After 10 min of incubation at room temperature, the absorbance at 550 nm was measured. Levels of nitrite was extrapolated using the sodium nitrite standard curve.

### 2.8. Quantitative Real-Time PCR

Changes in the expression profile of TNF-*α* and type I IFNs were determined using real-time PCR. Total RNA was isolated from BMDM cells using the TRI Reagent, according to the manufacturer's instructions (Sigma-Aldrich). Thereafter, 1 *μ*g of total RNA was incubated with deoxyribonuclease I (Thermo Fisher Scientific) and reverse transcribed (RT) into cDNA using the M-MLV reverse transcriptase (Promega). cDNA was subjected to qPCR with GoTaq qPCR Master Mix with BRYT Green dye (Promega) on a real-time PCR system (CFX Connect Real-Time System, Bio-Rad). For the amplification of the specific genes, the following primers were used: *Ifnβ*, forward: 5′-AACTTCCAAAACTGAAGACC-3′ and reverse: 5′-AACTCTGTTTTCCTTTGACC-3′; *Tnf-α*, forward: 5′-CTATGTCTCAGCCTCTTCTC-3′ and reverse: 5′-CATTTGGGAACTTCTCATCC-3′. For each mRNA quantification, the housekeeping gene HPRT (hypoxanthine phosphoribosyltransferase 1) was used as a reference point using the following primer: *Hprt*, forward: 5′-AGGGATTTGAATCACGTTTG-3′ and reverse: 5′-TTTACTGGCAACATCAACAG-3′. Real-time PCR data were analysed using the 2^–ΔΔCT^ method.

### 2.9. Assay for TNF-*α* Secretion

BMDM cells (1 × 10^6^/ml) were distributed in duplicate into 24-well flat-bottomed tissue culture plates and cultured overnight in Dulbecco's modified medium. Then, cells were treated with yolkin at doses ranging from 10 to 150 *μ*g/ml, as a potential inducer of TNF-*α*. LPS (1 *μ*g/ml) was used as a control activator of macrophages, while ovalbumin (100 *μ*g/ml) was used as a negative control. After 24 hours of stimulation, the level of TNF-*α* in supernatants was determined by ELISA.

### 2.10. Assay for Type I Interferon Secretion

BMDM cells (3 × 10^4^ cells per well) were placed in a 96-well plate and cultured overnight in Dulbecco's modified medium. Then, cells were treated with yolkin at doses ranging from 10 to 150 *μ*g/ml, as a potential inducer of type I interferons (IFN-*α*/*β*). LPS (1 *μ*g/ml) was used as a control activator of macrophages, while ovalbumin (100 *μ*g/ml) was used as a negative control. After 16 hours of stimulation, the level of biologically active type I interferons was determined by a bioassay.

### 2.11. Measurement of Biologically Active Type I Interferon Level by Bioassay

The level of biologically active type I interferons was determined by a bioassay using the B16-Blue™ IFN-*α*/*β* cell line according to the manufacturer's instruction (InvivoGen, San Diego, CA, USA). Briefly, 180 *μ*l of B16-Blue™ IFN-*α*/*β* cell suspension (4.2 × 10^5^ cells/ml) was placed in a 96-well plate and 20 *μ*l of each sample of the BMDM cell supernatant was added and then incubated at 37°C in 5% CO_2_ for 20 hours. 20 *μ*l of 10 *μ*g/ml poly(I : C) was used as a positive test control, while 20 *μ*l of test medium was used as a negative control. Next day, 180 *μ*l of QUANTI-Blue™ medium was placed in a 96-well plate and 20 *μ*l of each sample of the B16-Blue™ IFN-*α*/*β* cell supernatant was added and then incubated at 37°C for 5 hours. After this time, the absorbance at 655 nm was measured.

### 2.12. Measurement of TNF-*α* Level by ELISA

TNF-*α* secreted from BMDM cells were determined by an enzyme-linked immunosorbent assay (ELISA) using the Mouse TNF-*α* ELISA Max™ Deluxe Kit (BioLegend, San Diego, CA, USA) according to the procedure recommended by the manufacturer.

### 2.13. Western Blotting

BMDM cells (1 × 10^6^ cells/ml) were seeded onto poly-L-lysine-coated 6-well culture plates and incubated for 0 to 90 min with yolkin (10–150 *μ*g/ml) for ERK 1/2 and JNK kinase activation or for 24 h for TLR4 and iNOS expression. To determine the impact of ERK 1/2 and JNK kinases on the regulation of iNOS expression, BMDM cells were pretreated for 2 h with the selective kinase inhibitors U0126 (for ERK1/2) and SP600125 (for JNK) and then stimulated with yolkin (100 *μ*g/ml) for 24 h. After stimulation, the cells were lysed in RIPA buffer (150 mM NaCl; 50 mM Tris-HCl, pH 7.5; 5 mM EDTA; 1% Triton X-100; 0.1% SDS; and 0.5% deoxycholate) supplemented with protease inhibitor cocktail (Roche), 1 mM NaF, and 2 mM Na_3_VO_4_ on ice for 30 min. Lysates were centrifuged at 14,000 x g for 10 min at 4°C, and then protein content was determined by the bicinchoninic acid method using BSA as a standard. 30 *μ*g of protein samples were separated on 12% sodium dodecyl sulfate- (SDS-) polyacrylamide gel and next transferred to a nitrocellulose membrane. The membrane was blocked (Tris-HCl buffer, pH 7.0; 5% Tween 20 (TBST); and 5% nonfat dried milk) for 1 h at room temperature, then probed overnight at 4°C with the following primary antibodies diluted in TBST with 5% BSA: anti-iNOS (1 : 1000), anti *β*-actin (1 : 1000), anti-TLR4 (1 : 1000), anti-ERK 1/2 (1 : 2000), anti-phospho-ERK 1/2 (1: 2000), anti-JNK (1 : 2000), or anti-phospho-JNK (1 : 2000). Next, the membrane was incubated for 1 h at room temperature using secondary antibodies conjugated with alkaline phosphatase (1 : 10000 in TBST with 5% BSA) according to the standard procedure. Immunocomplexes were visualized using a NBT/BCIP substrate and analysed in the Molecular Imager ChemiDoc MP Imaging System (Bio-Rad, CA, USA).

### 2.14. Measurement of Antiviral Activity of Yolkin

#### 2.14.1. Virus and Cell Line

A wild-type Indiana VSV (*vesicular stomatitis virus*, *Rhabdoviridae*) serotype was originally obtained from Dr. C. Buckler (National Institutes of Health, Bethesda, MD, USA). VSV was grown and titrated in L_929_ cells. The titer was expressed with reference to the TCID_50_ (tissue culture infectious dose) value, based on the cytopathic effect caused by this virus in approximately 50% of infected cells.

L_929_ (ATCC CCL1), a murine fibroblast-like cell line, was maintained in complete RPMI 1640 medium (IIET, Wroclaw, Poland) with antibiotics (100 U/ml penicillin and 100 *μ*g/ml streptomycin), 2 mM/L-glutamine, and 10% fetal bovine serum (FBS) (all from Sigma-Aldrich, USA).

#### 2.14.2. Modified Antiviral Assay (AVA)

**Scheme 1:**
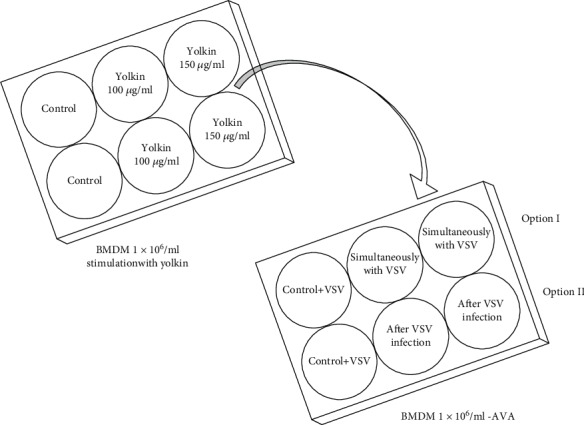
Antiviral activity of yolkin. Scheme of modified AVA.

A 24-hour culture of BMDM with a density of 1 × 10^6^/ml carried out on a 6-well plate was treated with yolkin at concentrations of 100 *μ*g/ml and 150 *μ*g/ml. The negative control were cells untreated with yolkin. After 24 h of incubation, medium above the cells was collected for AVA. Evaluation of the presence of antiviral molecules, i.e., IFN in this medium, was investigated. AVA was performed on BMDM cells. For this purpose, an additional BMDM culture at a density of 1 × 10^6^/ml was maintained on a 6-well plate. AVA was carried out in two options (Scheme 1):

*(1) Option I.* Medium above BMDM cells stimulated with yolkin at concentrations of 100 *μ*g/ml and 150 *μ*g/ml was added to additional BMDM cells simultaneously with a VSV with a dose 100 TCID_50_/ml (at a proportion of 1 : 1 with culture medium+10% HEPES+4.5 g glucose).

*(2) Option II.* Medium above BMDM cells stimulated with yolkin at concentrations of 100 *μ*g/ml and 150 *μ*g/ml was added to additional BMDM cells after VSV infection with a dose 100 TCID_50_/ml (at a proportion of 1 : 1 with culture medium+10% HEPES+4.5 g glucose).

Negative control were BMDM infected with VSV with a dose 100 TCID_50_/ml cells and untreated with medium after yolkin stimulation. After 24 hours of incubation, supernatants above BMDM were collected to determine the viral titer. All experimental options were carried out 3 times for 3 repetitions each. The VSV titer was finally determined based on the TCID_50_ method on the L_929_ culture.

### 2.15. Data/Statistical Analysis

Statistical analysis was performed using the software package Statistica 6 by StatSoft. Results are presented as median ± quartiles (25%-75%) and Min–Max or mean ± SD. Data were analysed using either Student's *t*-test or nonparametric Wilcoxon test. A value of *p* ≤ 0.05 was considered statistically significant.

## 3. Results

### 3.1. Characterisation of Yolkin Preparation

**Figure 1:**
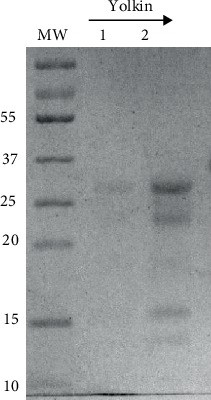
SDS-PAGE analysis of yolkin preparation. 10 *μ*g (line 1) and 20 *μ*g (line 2) of yolkin preparation were subjected to electrophoresis followed by staining with Coomassie Brilliant Blue R-250. MW: molecular weight marker (Thermo Fisher Scientific).

It was shown that yolkin preparation isolated from hen egg yolks using size-exclusion chromatography is free of bacterial endotoxins (data not shown). Electrophoretic analysis revealed that the yolkin preparation, which has been separated from the sample of IgY, is a mixture of several peptides of MW ranging from over 10 to about 35 kDa (Figure 1).

### 3.2. Effect of Yolkin on Cell Viability

**Figure 2:**
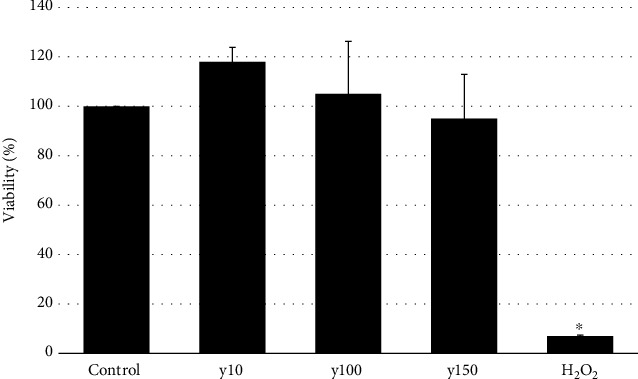
Effect of yolkin on the viability of bone marrow-derived macrophages (BMDM). BMDM cells (1 × 10^5^/ml) were exposed to yolkin (10, 100, and 150 *μ*g/ml) for 24 h. H_2_O_2_ (100 *μ*M) was used as a negative sample. Cell viability was evaluated by the MTT assay. The data are means ± S.D. of four independent experiments. ^∗^*p* ≤ 0.05 vs. control.

According to MTT assay results, it was shown that the yolkin polypeptide complex is not toxic for BMDM cells at doses ranging from 10 to 150 *μ*g/ml (Figure 2).

### 3.3. Yolkin Upregulates Innate Immune Response in BMDM Cells

The first objective of our study was to determine the impact of the yolkin polypeptide complex on the activation of BMDM cells. Therefore, the cells were incubated with different concentrations of yolkin. Next, the supernatants were removed and used to determine proinflammatory mediators such as cytokines (TNF-*α*, type I interferons) and nitric oxide.

LPS from *E. coli* (serotype O55 : B5) was used in this study only as a control activator of BMDM macrophages, which is able to trigger TLR4-dependent signaling pathways providing upregulation of expression of inflammatory mediators such as nitric oxide or cytokines.

#### 3.3.1. Yolkin Induces Nitric Oxide Production

**Figure 3:**
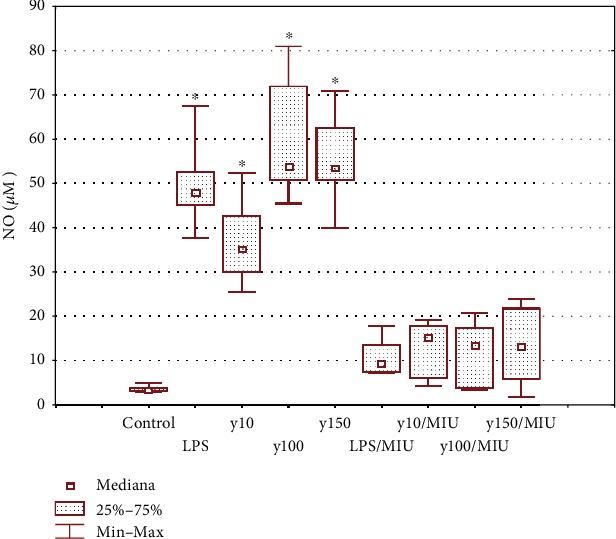
Effect of yolkin on nitric oxide production in BMDM cells. BMDM cells (1 × 10^6^/ml) were treated with yolkin (10, 100, and 150 *μ*g/ml) or LPS (1 *μ*g/ml) in the presence or absence of an S-MIU inhibitor (10 *μ*M) for 24 h. Thereafter, supernatants were collected and the level of NO was detected by the Griess reaction. The difference in the level of NO in control and yolkin-treated cells was significant (^∗^*p* ≤ 0.05). Results represent three independent experiments, and data are presented here as median ± Min–Max.

**Figure 4:**
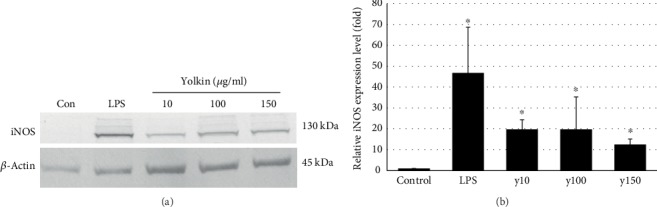
Upregulation of iNOS expression in BMDM cells after yolkin treatment. BMDM cells were treated with yolkin (10, 100, and 150 *μ*g/ml) or LPS (1 *μ*g/ml) or left untreated for 24 h. The level of iNOS protein was detected in cell lysates by immunoblotting using monoclonal anti-iNOS antibodies (a). Fold change in iNOS levels compared to *β*-actin (b). Results represent 3-4 independent experiments, and data are presented as mean ± SD. ^∗^*p* ≤ 0.05 vs. control.

Nitric oxide level was determined in supernatants of BMDM cells treated with yolkin preparation using the Griess reaction. As it was shown in Figure 3, yolkin upregulates NO production and this effect was dose dependent. An increase of the concentration of yolkin from 10 to 150 *μ*g/ml resulted in a significant improvement in the level of released NO (*p* ≤ 0.05), from 34 *μ*M to 53 *μ*M, respectively. The induction of NO production observed in response to yolkin was abolished by the selective iNOS inhibitor, S-MIU (Figure 3). NO production via upregulation of iNOS expression plays an essential role in the activation of immune response [16, 17]. Therefore, the impact of yolkin on iNOS expression was determined by Western blotting. As it was presented in Figure 4, yolkin significantly increased iNOS expression in BMDM cells. An approximately 20-fold increase in the iNOS level in yolkin-treated cells compared to control nontreated cells was observed.

#### 3.3.2. Yolkin Induces Type I Interferons and TNF-*α* Expression and Production

**Figure 5:**
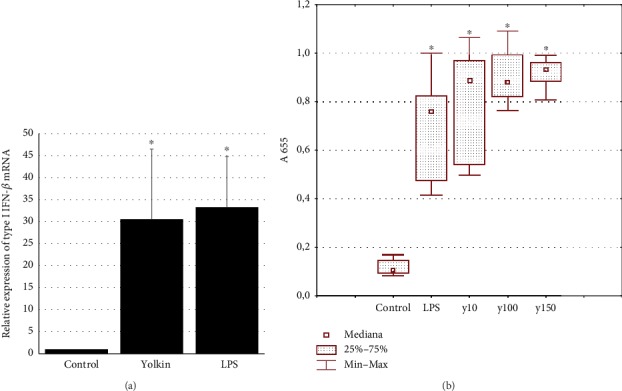
Upregulation of type I interferon expression (a) and production (b) after yolkin treatment of BMDM cells. (a) BMDM cells (1 × 10^6^/ml) were cultured alone or stimulated with yolkin (100 *μ*g/ml) or with LPS (1 *μ*g/ml). After 4 h of stimulation, total RNA was isolated from the cells and expression of type I IFN mRNA was determined by real-time PCR. (b) BMDM cells (1 × 10^6^/ml) were either treated with yolkin (10, 100, and 150 *μ*g/ml) or LPS (1 *μ*g/ml) or left untreated for 24 h. Supernatants were collected, and the level of type I interferons was determined by bioassay. Results are presented as mean ± SD (*n* = 3‐4). ^∗^*p* ≤ 0.05: statistically significant difference in value versus control.

**Figure 6:**
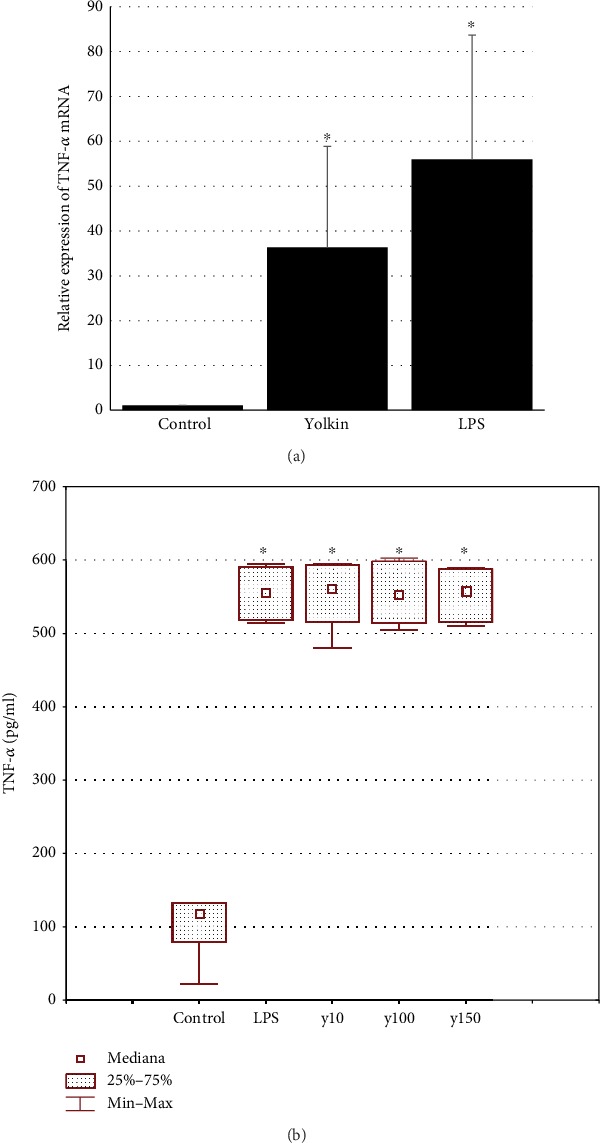
Upregulation of TNF-*α* expression (a) and production (b) after yolkin treatment of BMDM cells. (a) BMDM cells (1 × 10^6^/ml) were cultured alone or stimulated with yolkin (100 *μ*g/ml) or with LPS (1 *μ*g/ml). After 4 h of stimulation, total RNA was isolated from the cells and expression of TNF-*α* mRNA was determined by real-time PCR. (b) BMDM cells (1 × 10^6^/ml) were either treated with yolkin (10, 100, and 150 *μ*g/ml) or LPS (1 *μ*g/ml) or left untreated for 24 h. Supernatants were collected, and the level of TNF-*α* was determined by ELISA. Results are presented as mean ± SD (*n* = 3‐4). ^∗^*p* ≤ 0.05: statistically significant difference in value versus control.

To determine whether the polypeptide complex yolkin activates IFNs (*α*/*β*) and TNF-*α* expression and production, BMDM cells were treated with yolkin at doses from 10 to 150 *μ*g/ml. Changes in cytokine expression were determined by real-time PCR, while the levels of type I interferons and TNF-*α* were determined by bioassay and ELISA, respectively. Our data demonstrated that 16-24 h of yolkin treatment enhances IFN-*β* (Figure 5(a)) and TNF-*α* (Figure 6(a)) expression and IFN (*α*/*β*) (Figure 5(b)) and TNF-*α* production (Figure 6(b)), compared to untreated control cells. It was observed that yolkin significantly increased both IFN and TNF-*α* expression and production at doses over 10 *μ*g/ml. Proinflammatory cytokine production by BMDM cells treated with 150 *μ*g/ml of yolkin was approximately 9-fold higher for IFNs (*α*/*β*), and about 6-fold higher for TNF-*α*, compared to nontreated control cells.

### 3.4. TLR4 and MAPK Signaling Pathways Are Involved in Yolkin-Induced Macrophage Activation

The results mentioned above showed that yolkin is capable of inducing the expression and production of inflammatory mediators, such as nitric oxide TNF-*α* and type I interferons.

Once the effect of yolkin on BMDM macrophages was established, our next objective was to find out what are the mechanisms underlying the abovementioned observations.

Firstly, we used BMDM TLR4-deficient cells to elaborate the role of TLR4 in yolkin-induced macrophage activation. BMDM TLR4-deficient cells were incubated with yolkin (10 to 150 *μ*g/ml), as a potential inducer of nitric oxide. LPS (1 *μ*g/ml), a classical inducer of TLR4 receptor, was used as a control TLR4 agonist. It was observed that macrophages lacking the TLR4 receptor did not respond to yolkin (and also LPS) and were unable to produce nitric oxide (data not shown).

Secondly, the impact of yolkin on TLR4 expression in BMDM macrophages was determined by Western blotting. However, no significant changes in TLR4 level was observed (data not shown).

**Figure 7:**
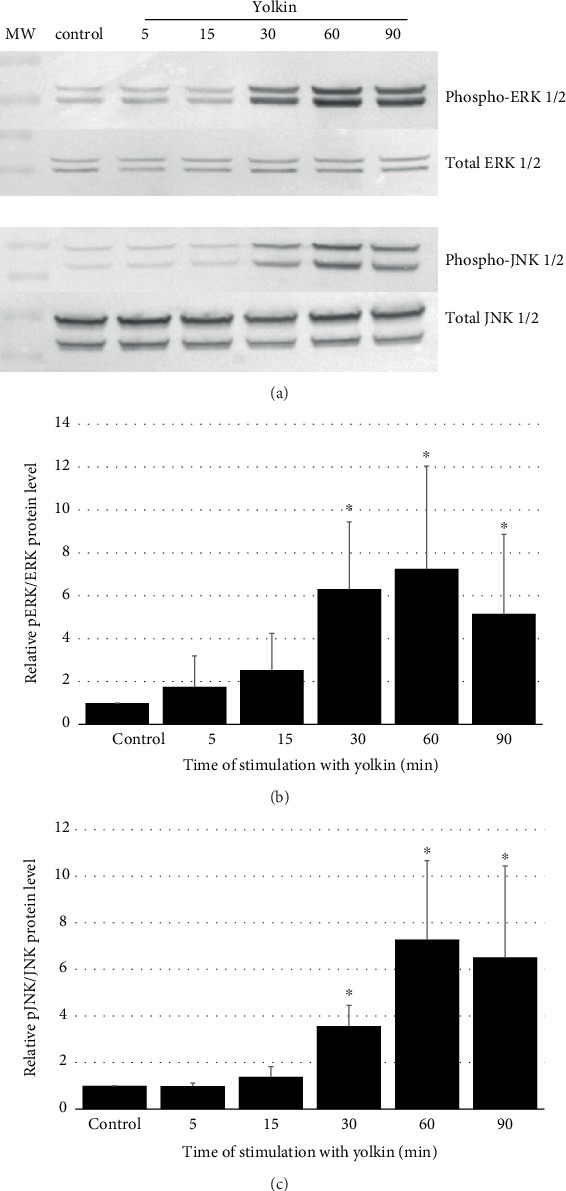
Effect of yolkin treatment on MAPK activation in BMDM cells. BMDM cells were treated with yolkin (100 *μ*g/ml) for 0, 5, 15, 30, 60, and 90 min at 37°C. Next, cells were lysed and subjected to SDS-PAGE followed by Western blotting using monoclonal antibody against total ERK, phospho-ERK 1/2, total JNK, and phospho-JNK 1/2 (a). Data were expressed as a fold of change in density compared with control cells using Molecular Imager ChemiDoc MP Imaging System with ImageLab Software (b, c) (Bio-Rad) (Student's *t*-test, mean ± SD of *n* = 4‐5). ^∗^*p* ≤ 0.05 compared to control cells.

We also decided to find out what was the effect of yolkin on the activation of MAP kinase signaling pathways responsible for the regulation of cytokine and iNOS expression. BMDM cells were treated with yolkin for 0 to 90 min, and the level of phosphorylated and total MAPK, i.e., ERK 1/2 and JNK, were determined by Western blotting. It was shown that after 15 min of yolkin treatment, a significant increase in MAPK phosphorylation was observed and after 90 min this declined to control level (Figure 7). Both kinases ERK 1/2 and JNK may participate in the regulation of expression of proinflammatory cytokines and iNOS.

**Figure 8:**
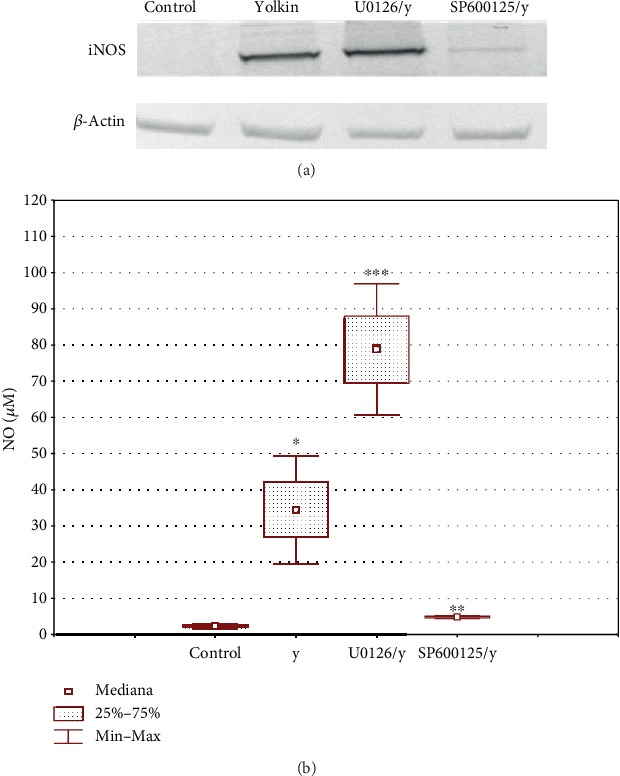
Effect of MAPK inhibitors on yolkin-mediated iNOS expression (a) and activation (b). BMDM cells were preincubated with MAPK inhibitors: U0126 for ERK and SP600125 for JNK for 2 h, followed by treatment with yolkin. Cells were lysed and expression of iNOS was determined by Western blot using monoclonal anti-iNOS antibodies (a). Supernatants were used to determine NO concentration by the Griess reaction (b). Results represent three independent experiments and present mean ± SD (*n* = 3). ^∗^*p* ≤ 0.05 compared with the control, and ^∗∗^*p* ≤ 0.05 and ^∗∗∗^*p* ≤ 0.05 compared with yolkin.

Next, we investigated the role of MAPK in yolkin-mediated iNOS expression in macrophages using the following pharmacological inhibitors of MAP kinases: U0126 for ERK1/2 and SP600125 for JNK. The cells were firstly pretreated with inhibitors for 2 hours and next incubated with yolkin for 24 hours. The results showed that only JNK inhibitor SP600125 significantly decreased yolkin-induced iNOS expression in BMDM macrophages (Figure 8(a)), whereas a significant increase of yolkin-induced iNOS expression was observed after ERK 1/2 inhibition. We also observed a significant reduction in the NO level in response to yolkin in SP600125-pretreated cells and NO overproduction in response to yolkin in U0126-pretreated macrophages (Figure 8(b)). These findings suggest that the JNK pathway is related to yolkin-induced iNOS expression; however, yolkin-dependent ERK 1/2 activation, described previously, can be connected with the upregulation of the TNF-*α* expression.

### 3.5. Yolkin as a Potential Antiviral Agent

**Figure 9:**
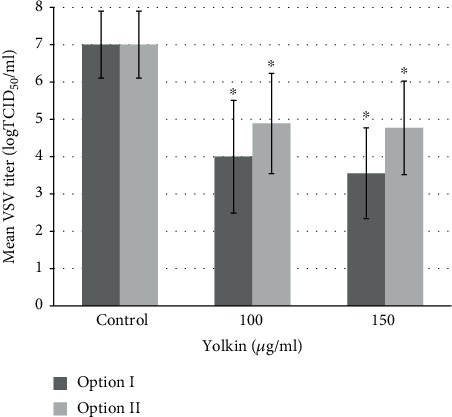
Evaluation of the antiviral activity of yolkin in the BMDM mouse macrophage model. A 24 hour preincubation of BMDM cells with yolkin at concentrations of 100 *μ*g/ml and 150 *μ*g/ml stimulates cells to produce type I IFNs which in turn inhibits VSV replication. Option I—medium above BMDM cells stimulated with yolkin at concentrations of 100 *μ*g/ml and 150 *μ*g/ml was added to additional BMDM cells simultaneously with VSV with a dose of 100 TCID_50_/ml. Option II—medium above BMDM cells stimulated with yolkin at concentrations of 100 *μ*g/ml and 150 *μ*g/ml was added to additional BMDM cells after VSV infection with a dose of 100 TCID_50_/ml.

Yolkin expresses an antiviral activity in the BMDM mouse macrophage model infected with VSV. Yolkin at concentrations of 100 *μ*g/ml and 150 *μ*g/ml stimulated production of type I IFNs that inhibit VSV replication. A dose-dependent decrease in an average virus titer compared to control in Option I is shown. After stimulation with yolkin, the following maximum VSV titer reductions in BMDM cells treated with medium were observed: for yolkin at a concentration of 150 *μ*g/ml—virus reduction of 3.44 log, and for yolkin at a concentration of 100 *μ*g/ml—virus reduction of 3 log. A decrease in the virus titer over 3 log indicates the decrease of infectivity by more than 99.9%. There was no dose-dependent decrease in VSV replication in BMDM cells in Option II; however, the antiviral activity of yolkin was demonstrated. Maximum VSV titer reduction in BMDM cells treated with medium after stimulation with yolkin (Option II) at the concentration of 150 *μ*g/ml and 100 *μ*g/ml resulted in about 2 log. Data are presented in Figure 9.

## 4. Discussion

Naturally occurring bioactive compounds present in human diet have utmost significance in promoting and maintaining health, especially those with high potency and low toxicity to normal cells [20–22]. Egg yolk represents a major source of active principles, usable in medical, pharmaceutical, or nutraceutical industries [2, 20]. It was shown by Polanowski et al. [6, 7] that hen egg yolk immunoglobulin Y occurs as a complex with polypeptides, named yolkin, which are homologous with the C-terminal domain of vitellogenin II. In our previous studies, we reported that yolkin is a good immunoregulator and stimulates human whole blood cells to produce significant amounts of cytokines [6–8]. In the present study, we explained the mechanism of action of the yolkin polypeptide complex in murine bone marrow-derived macrophages (BMDM cell line).

Search of new, more effective natural compounds that can potentiate immunological response for immunopharmacological purposes is very important [21–23]. Stimulation of innate immune response is regarded as one of the important strategies to enhance the body's defense systems, especially in the elderly and in cancer patients. It has been shown in rodent models that advanced age is associated with diminished response to pathogens and lower level production of the proinflammatory mediators compared to young controls [11, 24, 25]. Several studies also supported a reduction in the production of cytokines (like TNF-*α* or IFN-*γ*), NO, or macrophage inflammatory protein-1*α* (CCL3) in response to different stimuli [25, 26]. Macrophages play pivotal roles in modulating immune function. They maintain homeostasis, protect against tumors and bacterial and viral infection, and secrete numerous particles to regulate the activity of other cells [14, 27]. Age-related changes in macrophage function have been correlated with susceptibility to infection and weaker response to injury. Hence, studies undertaken with macrophages may be good indicators of immune status. Macrophages can be activated through members of the Toll-like receptor (TLR) family, such as TLR4 [15]. Classically activated macrophages play a crucial role in nonspecific immune response by the production of a wide spectrum of reagents. The major mediators of macrophage effector function, representing the proinflammatory M1 macrophage profile, is the generation of cytokines (e.g., IFNs, TNF-*α*, and IL-6), chemokines, reactive oxygen species (ROS), and also nitric oxide (NO) [14, 21, 28]. NO exerts beneficial results for the body by playing an important role as an antibacterial, antiparasitic, antitumor, and antiviral agent [29–31], while TNF-*α* and type I interferons are the major proinflammatory cytokines produced by macrophages in response to pathogens. Type I interferons (IFN-*α*/*β*) are anti-viral cytokines critical to the host immune response against viruses [32].

In this study, we showed that macrophages of the murine BMDM cell line are activated by the yolkin polypeptide complex resulting in the upregulation of the expression of iNOS synthase and the production of significant (but not toxic for themselves) amounts of NO and proinflammatory cytokines. This effect was dose dependent and was inhibited by a selective iNOS inhibitor (S-MIU). We also checked if yolkin can enhance the mRNA expression and protein production of the following proinflammatory cytokines: TNF-*α* and type I interferons. The significant increase in TNF-*α* and type I interferon expression and production were observed in BMDM cells, thus enhancing the microbicidal properties of the macrophages. Some studies have shown that the production of nitric oxide by classically activated macrophages plays a critical role in the intracellular clearance of pathogens [33–35]. Additionally, activated macrophages producing proinflammatory cytokines like TNF-*α* or IFN-*α*/*β* or IL-6 exhibit an antitumor response [36]. It was also important to check what were the potential mechanisms activated by yolkin that trigger the activation of BDMD secretory activity. One of them was the possible ability of yolkin to bind and activate the TLR4 receptor. It was observed that TLR4-deficient BMDM cells incubated with yolkin did not produce nitric oxide. These results suggest that yolkin may act as a potential TLR4 agonist. Signaling through the TLR4 receptor triggers the activation of MAP kinases, such as ERK 1/2, JNK 1/2, and p38, and subsequently provides for the activation of transcriptional factors controlling the transcription of specific genes [37, 38]. In the present study, we have shown that the yolkin complex upregulates the phosphorylation of ERK 1/2 and JNK in BMDM cells, leading to their activation. It is well known that inducible nitric oxide synthase expression is regulated due to the activation of MAPKs, which leads to the phosphorylation of several transcription factors such as, e.g., nuclear factor kappa B (NF-*κ*B) or activating protein 1 (AP-1), which control the expression of target genes coding proinflammatory cytokines and iNOS [39–41]. BMDM cells were preincubated with pharmacological inhibitors of MAP kinases (i.e., U0126 (MEK 1/2 inhibitor) and SP600125 (JNK inhibitor)) to inhibit kinase activity, and subsequently, the impact of yolkin on the upregulation of iNOS expression and NO production was determined. The obtained data indicated that the inhibition of JNK kinase abolished iNOS expression and NO production in BMDM cells incubated with yolkin. It suggests that JNK plays an essential role in yolkin-induced iNOS expression and NO production in macrophages. In contrast to JNK, the inhibition of the ERK 1/2 signaling pathway using the U0126 inhibitor enhanced yolkin-induced upregulation of iNOS expression and NO production in macrophages [42]. It suggests that ERK kinases play a negative regulatory role in yolkin-dependent iNOS expression and NO production. A similar observation was shown by Bhatt et al. [42] in peptidoglycan-activated macrophages.

Many natural compounds, e.g. flavonoids, have been reported to upregulate the secretion of proinflammatory cytokines and iNOS in macrophages by the induction of MAPK phosphorylation and the activation of the NF-*κ*B transcription factor [43, 44]. Several reports have also demonstrated that polysaccharides isolated from different sources activate macrophages through TLR4-dependent signaling pathways to produce proinflammatory mediators [45]. Comparable immunoregulatory activity was also observed for the proline-rich polypeptide complex (PRP) isolated from ovine colostrum [46, 47]. Immunostimulatory activity of yolkin indicates that it is a bioavailable complex. However, the mechanism of the import of yolkin peptides into cells is, as yet, still unresolved.

The results of this study revealed that the yolkin polypeptide complex stimulates macrophage-derived NO, TNF-*α*, and type I IFN production and might be used as a potential immunostimulator for enhancing the early innate immune response. These properties may provide a mechanistic basis for antibacterial, antitumor, and antiviral activities and may activate certain functions of immune cells. While the type I IFNs have a diverse role, they are also essential for eliminating viral infections [32]. Also, NO can trigger different effector mechanisms in immune responses like the modification of host and viral molecules by nitrosylation [31]. Based on our results, we propose a possible mechanism of the antiviral effect of yolkin by indirect action, when antiviral response depends on proinflammatory mediators produced by yolkin-activated macrophages. Our data showed no direct effect of yolkin on the multiplication of VSV. However, a significant decrease of the VSV titer was observed after 24 h of incubation of VSV-infected BMDM cells with supernatants obtained from BMDM macrophages previously treated with yolkin. It suggests that the concentrations of reactive nitrogen intermediates, TNF-*α* and type I IFNs (*α*/*β*) presented in supernatants, are sufficient to trigger an antiviral response.

## 5. Conclusion

Our study reveals that the yolkin polypeptide complex isolated from hen egg yolk activates macrophages of the BMDM cell line to express/produce innate immunity mediators such as TNF-*α*, type I interferons, and NO, which are important in the regulation of an innate immune response against pathogens or cancer cells. Additionally, yolkin modulates the activity of the JNK and ERK 1/2 kinases which control the expression of the abovementioned proinflammatory mediators. The upregulation of the innate response is regarded as one of the important strategies to enhance the body's defense systems, especially in the elderly, because advanced age is associated with a diminished response to different stimulators and production of insufficient amounts of the proinflammatory mediators. It is also important in cancer treatment, where chemotherapy and radiation are always accompanied by immunosuppression. The ability of yolkin to activate macrophages of the proinflammatory type (M1), as demonstrated here, may provide a mechanistic basis for the activation of certain functions of immune cells and give promising possibilities for safe therapeutic intervention.

## Data Availability

The data that support the findings of this study are available from the corresponding author upon reasonable request.
